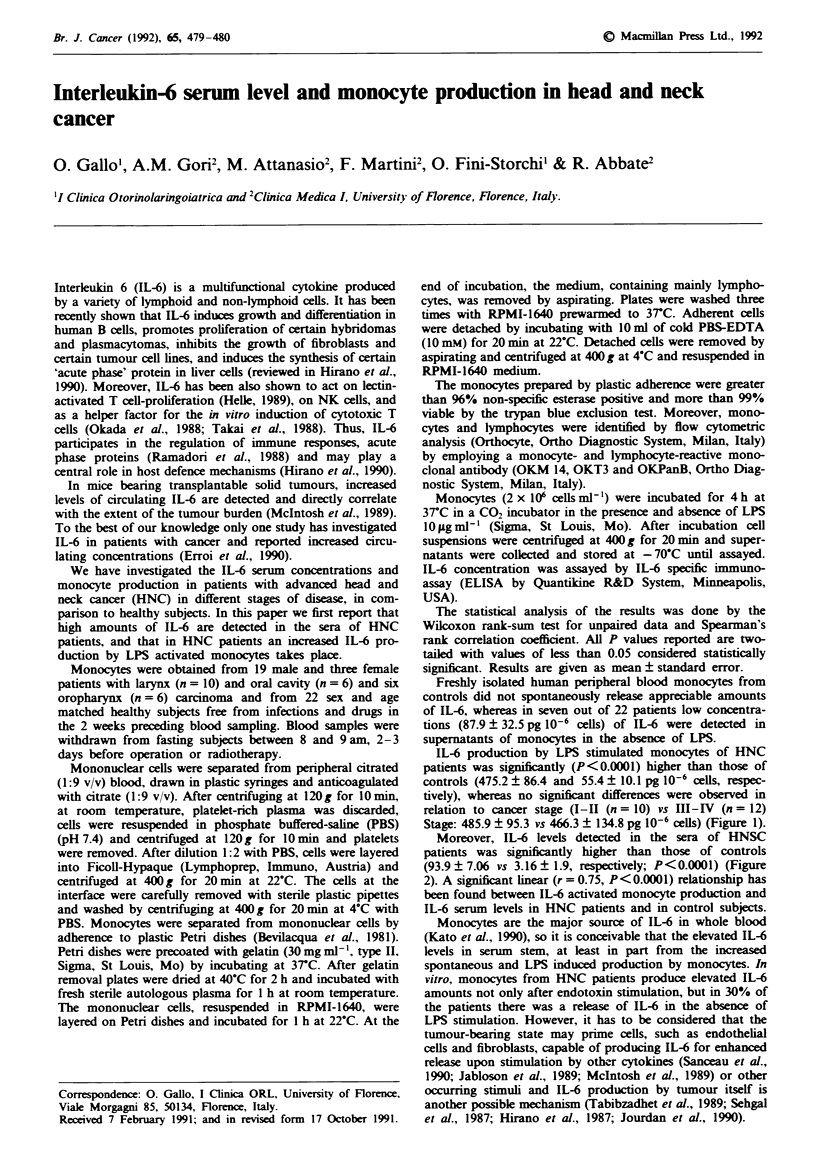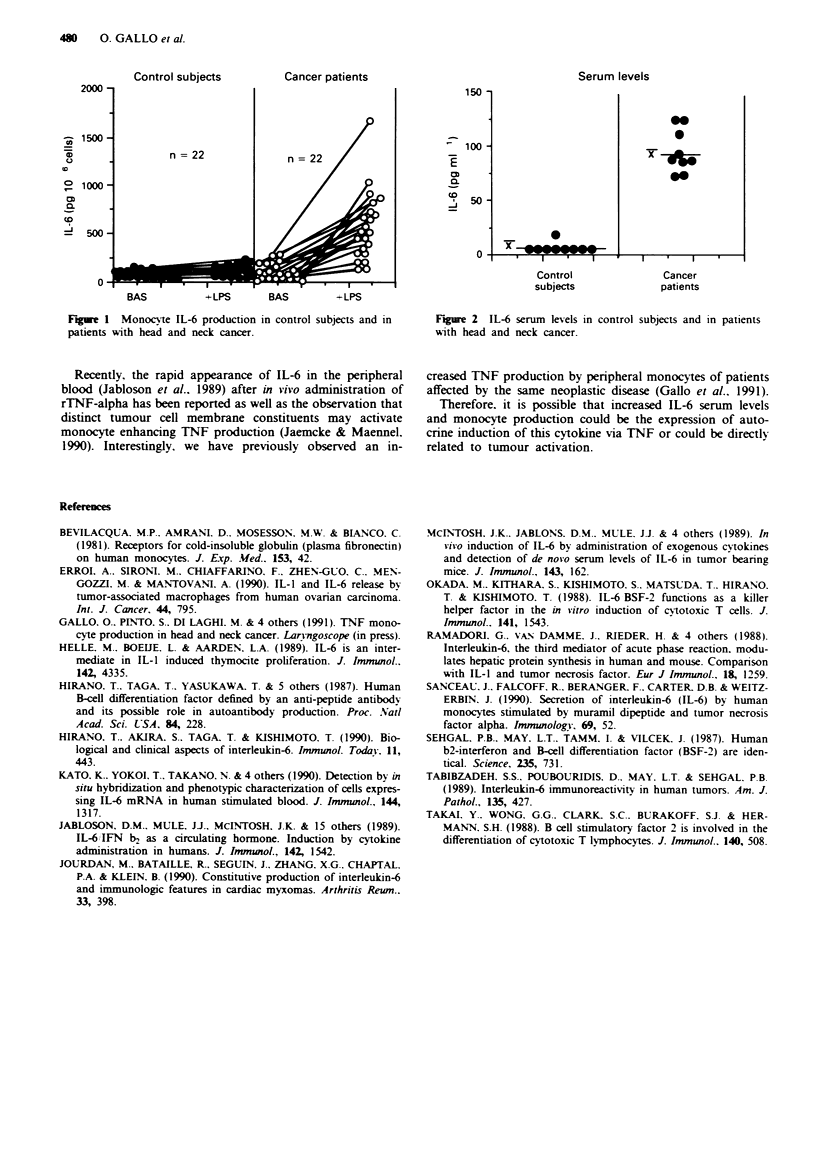# Interleukin-6 serum level and monocyte production in head and neck cancer.

**DOI:** 10.1038/bjc.1992.97

**Published:** 1992-03

**Authors:** O. Gallo, A. M. Gori, M. Attanasio, F. Martini, O. Fini-Storchi, R. Abbate

**Affiliations:** I Clinica Otorinolaringoiatrica, University of Florence, Italy.


Br. J. Cancer (1992), 65, 479-480 © Macmillan Press Ltd., 1992

### Interleukin-6 serum level and monocyte production in head and neck cancer

O. Gallo[1], A.M. Gori[2], M. Attanasio[2], F. Martini[2], O. Fini-Storchi[1] & R. Abbate[2]

[1]*I Clinica Otorinolaringoiatrica and* [2]*Clinica Medica I, University of Florence, Florence, Italy.*

Interleukin 6 (IL-6) is a multifunctional cytokine produced by a variety of lymphoid and non-lymphoid cells. It has been recently shown that IL-6 induces growth and differentiation in human B cells, promotes proliferation of certain hybridomas and plasmacytomas, inhibits the growth of fibroblasts and certain tumour cell lines, and induces the synthesis of certain 'acute phase' protein in liver cells (reviewed in Hirano *et al.*, 1990). Moreover, IL-6 has been also shown to act on lectin-activated T cell-proliferation (Helle, 1989), on NK cells, and as a helper factor for the *in vitro* induction of cytotoxic T cells (Okada *et al.*, 1988; Takai *et al.*, 1988). Thus, IL-6 participates in the regulation of immune responses, acute phase proteins (Ramadori *et al.*, 1988) and may play a central role in host defence mechanisms (Hirano *et al.*, 1990).

In mice bearing transplantable solid tumours, increased levels of circulating IL-6 are detected and directly correlate with the extent of the tumour burden (McIntosh *et al.*, 1989). To the best of our knowledge only one study has investigated IL-6 in patients with cancer and reported increased circulating concentrations (Erroi *et al.*, 1990).

We have investigated the IL-6 serum concentrations and monocyte production in patients with advanced head and neck cancer (HNC) in different stages of disease, in comparison to healthy subjects. In this paper we first report that high amounts of IL-6 are detected in the sera of HNC patients, and that in HNC patients an increased IL-6 production by LPS activated monocytes takes place.

Monocytes were obtained from 19 male and three female patients with larynx ($n = 10$) and oral cavity ($n = 6$) and six oropharynx ($n = 6$) carcinoma and from 22 sex and age matched healthy subjects free from infections and drugs in the 2 weeks preceding blood sampling. Blood samples were withdrawn from fasting subjects between 8 and 9 am, 2-3 days before operation or radiotherapy.

Mononuclear cells were separated from peripheral citrated (1:9 v/v) blood, drawn in plastic syringes and anticoagulated with citrate (1:9 v/v). After centrifuging at 120 *g* for 10 min, at room temperature, platelet-rich plasma was discarded, cells were resuspended in phosphate buffered-saline (PBS) (pH 7.4) and centrifuged at 120 *g* for 10 min and platelets were removed. After dilution 1:2 with PBS, cells were layered into Ficoll-Hypaque (Lymphoprep, Immuno, Austria) and centrifuged at 400 *g* for 20 min at 22°C. The cells at the interface were carefully removed with sterile plastic pipettes and washed by centrifuging at 400 *g* for 20 min at 4°C with PBS. Monocytes were separated from mononuclear cells by adherence to plastic Petri dishes (Bevilacqua *et al.*, 1981). Petri dishes were precoated with gelatin (30 mg ml$^{-1}$, type II, Sigma, St Louis, Mo) by incubating at 37°C. After gelatin removal plates were dried at 40°C for 2 h and incubated with fresh sterile autologous plasma for 1 h at room temperature. The mononuclear cells, resuspended in RPMI-1640, were layered on Petri dishes and incubated for 1 h at 22°C. At the end of incubation, the medium, containing mainly lymphocytes, was removed by aspirating. Plates were washed three times with RPMI-1640 prewarmed to 37°C. Adherent cells were detached by incubating with 10 ml of cold PBS-EDTA (10 mM) for 20 min at 22°C. Detached cells were removed by aspirating and centrifuged at 400 *g* at 4°C and resuspended in RPMI-1640 medium.

The monocytes prepared by plastic adherence were greater than 96% non-specific esterase positive and more than 99% viable by the trypan blue exclusion test. Moreover, monocytes and lymphocytes were identified by flow cytometric analysis (Orthocyte, Ortho Diagnostic System, Milan, Italy) by employing a monocyte- and lymphocyte-reactive monoclonal antibody (OKM 14, OKT3 and OKPanB, Ortho Diagnostic System, Milan, Italy).

Monocytes ($2 \times 10^6$ cells ml$^{-1}$) were incubated for 4 h at 37°C in a $CO_2$ incubator in the presence and absence of LPS 10 μg ml$^{-1}$ (Sigma, St Louis, Mo). After incubation cell suspensions were centrifuged at 400 *g* for 20 min and supernatants were collected and stored at −70°C until assayed. IL-6 concentration was assayed by IL-6 specific immunoassay (ELISA by Quantikine R&D System, Minneapolis, USA).

The statistical analysis of the results was done by the Wilcoxon rank-sum test for unpaired data and Spearman's rank correlation coefficient. All *P* values reported are two-tailed with values of less than 0.05 considered statistically significant. Results are given as mean ± standard error.

Freshly isolated human peripheral blood monocytes from controls did not spontaneously release appreciable amounts of IL-6, whereas in seven out of 22 patients low concentrations (87.9 ± 32.5 pg $10^{-6}$ cells) of IL-6 were detected in supernatants of monocytes in the absence of LPS.

IL-6 production by LPS stimulated monocytes of HNC patients was significantly ($P < 0.0001$) higher than those of controls (475.2 ± 86.4 and 55.4 ± 10.1 pg $10^{-6}$ cells, respectively), whereas no significant differences were observed in relation to cancer stage (I-II ($n = 10$) *vs* III-IV ($n = 12$) Stage: 485.9 ± 95.3 *vs* 466.3 ± 134.8 pg $10^{-6}$ cells) (Figure 1).

Moreover, IL-6 levels detected in the sera of HNSC patients was significantly higher than those of controls (93.9 ± 7.06 *vs* 3.16 ± 1.9, respectively; $P < 0.0001$) (Figure 2). A significant linear ($r = 0.75$, $P < 0.0001$) relationship has been found between IL-6 activated monocyte production and IL-6 serum levels in HNC patients and in control subjects.

Monocytes are the major source of IL-6 in whole blood (Kato *et al.*, 1990), so it is conceivable that the elevated IL-6 levels in serum stem, at least in part from the increased spontaneous and LPS induced production by monocytes. *In vitro*, monocytes from HNC patients produce elevated IL-6 amounts not only after endotoxin stimulation, but in 30% of the patients there was a release of IL-6 in the absence of LPS stimulation. However, it has to be considered that the tumour-bearing state may prime cells, such as endothelial cells and fibroblasts, capable of producing IL-6 for enhanced release upon stimulation by other cytokines (Sanceau *et al.*, 1990; Jabloson *et al.*, 1989; McIntosh *et al.*, 1989) or other occurring stimuli and IL-6 production by tumour itself is another possible mechanism (Tabibzadhet *et al.*, 1989; Sehgal *et al.*, 1987; Hirano *et al.*, 1987; Jourdan *et al.*, 1990).

Correspondence: O. Gallo, I Clinica ORL, University of Florence, Viale Morgagni 85, 50134, Florence, Italy.
Received 7 February 1991; and in revised form 17 October 1991.

**Figure 1** Monocyte IL-6 production in control subjects and in patients with head and neck cancer.

**Figure 2** IL-6 serum levels in control subjects and in patients with head and neck cancer.

Recently, the rapid appearance of IL-6 in the peripheral blood (Jabloson *et al.*, 1989) after *in vivo* administration of rTNF-alpha has been reported as well as the observation that distinct tumour cell membrane constituents may activate monocyte enhancing TNF production (Jaemcke & Maennel, 1990). Interestingly, we have previously observed an increased TNF production by peripheral monocytes of patients affected by the same neoplastic disease (Gallo *et al.*, 1991).

Therefore, it is possible that increased IL-6 serum levels and monocyte production could be the expression of autocrine induction of this cytokine via TNF or could be directly related to tumour activation.

### References

BEVILACQUA, M.P., AMRANI, D., MOSESSON, M.W. & BIANCO, C. (1981). Receptors for cold-insoluble globulin (plasma fibronectin) on human monocytes. *J. Exp. Med.*, **153**, 42.

ERROI, A., SIRONI, M., CHIAFFARINO, F., ZHEN-GUO, C., MENGOZZI, M. & MANTOVANI, A. (1990). IL-1 and IL-6 release by tumor-associated macrophages from human ovarian carcinoma. *Int. J. Cancer*, **44**, 795.

GALLO, O., PINTO, S., DI LAGHI, M. & 4 others (1991). TNF monocyte production in head and neck cancer. *Laryngoscope* (in press).

HELLE, M., BOEIJE, L. & AARDEN, L.A. (1989). IL-6 is an intermediate in IL-1 induced thymocite proliferation. *J. Immunol.*, **142**, 4335.

HIRANO, T., TAGA, T., YASUKAWA, T. & 5 others (1987). Human B-cell differentiation factor defined by an anti-peptide antibody and its possible role in autoantibody production. *Proc. Natl Acad. Sci. USA*, **84**, 228.

HIRANO, T., AKIRA, S., TAGA, T. & KISHIMOTO, T. (1990). Biological and clinical aspects of interleukin-6. *Immunol. Today*, **11**, 443.

KATO, K., YOKOI, T., TAKANO, N. & 4 others (1990). Detection by *in situ* hybridization and phenotypic characterization of cells expressing IL-6 mRNA in human stimulated blood. *J. Immunol.*, **144**, 1317.

JABLOSON, D.M., MULE, J.J., MCINTOSH, J.K. & 15 others (1989). IL-6/IFN $b_2$ as a circulating hormone. Induction by cytokine administration in humans. *J. Immunol.*, **142**, 1542.

JOURDAN, M., BATAILLE, R., SEGUIN, J., ZHANG, X.G., CHAPTAL, P.A. & KLEIN, B. (1990). Constitutive production of interleukin-6 and immunologic features in cardiac myxomas. *Arthritis Reum.*, **33**, 398.

MCINTOSH, J.K., JABLONS, D.M., MULE, J.J. & 4 others (1989). *In vivo* induction of IL-6 by administration of exogenous cytokines and detection of *de novo* serum levels of IL-6 in tumor bearing mice. *J. Immunol.*, **143**, 162.

OKADA, M., KITHARA, S., KISHIMOTO, S., MATSUDA, T., HIRANO, T. & KISHIMOTO, T. (1988). IL-6/BSF-2 functions as a killer helper factor in the *in vitro* induction of cytotoxic T cells. *J. Immunol.*, **141**, 1543.

RAMADORI, G., VAN DAMME, J., RIEDER, H. & 4 others (1988). Interleukin-6, the third mediator of acute phase reaction, modulates hepatic protein synthesis in human and mouse. Comparison with IL-1 and tumor necrosis factor. *Eur J Immunol.*, **18**, 1259.

SANCEAU, J., FALCOFF, R., BERANGER, F., CARTER, D.B. & WEITZERBIN, J. (1990). Secretion of interleukin-6 (IL-6) by human monocytes stimulated by muramil dipeptide and tumor necrosis factor alpha. *Immunology*, **69**, 52.

SEHGAL, P.B., MAY, L.T., TAMM, I. & VILCEK, J. (1987). Human b2-interferon and B-cell differentiation factor (BSF-2) are identical. *Science*, **235**, 731.

TABIBZADEH, S.S., POUBOURIDIS, D., MAY, L.T. & SEHGAL, P.B. (1989). Interleukin-6 immunoreactivity in human tumors. *Am. J. Pathol.*, **135**, 427.

TAKAI, Y., WONG, G.G., CLARK, S.C., BURAKOFF, S.J. & HERMANN, S.H. (1988). B cell stimulatory factor 2 is involved in the differentiation of cytotoxic T lymphocytes. *J. Immunol.*, **140**, 508.